# JC Polyomavirus Infection Is Strongly Controlled by Human Leucocyte Antigen Class II Variants

**DOI:** 10.1371/journal.ppat.1004084

**Published:** 2014-04-24

**Authors:** Emilie Sundqvist, Dorothea Buck, Clemens Warnke, Eva Albrecht, Christian Gieger, Mohsen Khademi, Izaura Lima Bomfim, Anna Fogdell-Hahn, Jenny Link, Lars Alfredsson, Helle Bach Søndergaard, Jan Hillert, International Multiple Sclerosis Genetics Consortium, Annette B. Oturai, Bernhard Hemme, Ingrid Kockum, Tomas Olsson

**Affiliations:** 1 Neuroimmunology Unit, Department of Clinical Neuroscience, Karolinska Institutet, Stockholm, Sweden; 2 Department of Neurology, Technische Universität München, Munich, Germany; 3 The Multiple Sclerosis Research Group, Department of Clinical Neuroscience, Karolinska Institutet, Stockholm, Sweden; 4 Institute of Genetic Epidemiology, Helmholtz Zentrum München - German Research Center for Environmental Health, Neuherberg, Germany; 5 Institute for Environmental Medicine, Karolinska Institutet, Stockholm, Sweden; 6 Danish Multiple Sclerosis Center, Department of Neurology, Copenhagen University Hospital, Rigshospitalet, Copenhagen, Denmark; 7 Munich Cluster for Systems Neurology (SyNergy), Munich, Germany; Brown University, United States of America

## Abstract

JC polyomavirus (JCV) carriers with a compromised immune system, such as in HIV, or subjects on immune-modulating therapies, such as anti VLA-4 therapy may develop progressive multifocal leukoencephalopathy (PML) which is a lytic infection of oligodendrocytes in the brain. Serum antibodies to JCV mark infection occur only in 50–60% of infected individuals, and high JCV-antibody titers seem to increase the risk of developing PML. We here investigated the role of human leukocyte antigen (HLA), instrumental in immune defense in JCV antibody response. Anti-JCV antibody status, as a surrogate for JCV infection, were compared to *HLA* class I and II alleles in 1621 Scandinavian persons with MS and 1064 population-based Swedish controls and associations were replicated in 718 German persons with MS. *HLA*-alleles were determined by SNP imputation, sequence specific (SSP) kits and a reverse PCR sequence-specific oligonucleotide (PCR-SSO) method. An initial GWAS screen displayed a strong *HLA* class II region signal. The HLA-DRB1*15 haplotype was strongly negatively associated to JCV sero-status in Scandinavian MS cases (OR = 0.42, p = 7×10^−15^) and controls (OR = 0.53, p = 2×10^−5^). In contrast, the DQB1*06:03 haplotype was positively associated with JCV sero-status, in Scandinavian MS cases (OR = 1.63, p = 0.006), and controls (OR = 2.69, p = 1×10^−5^). The German dataset confirmed these findings (OR = 0.54, p = 1×10^−4^ and OR = 1.58, p = 0.03 respectively for these haplotypes). HLA class II restricted immune responses, and hence CD4+ T cell immunity is pivotal for JCV infection control. Alleles within the HLA-DR1*15 haplotype are associated with a protective effect on JCV infection. Alleles within the DQB1*06:03 haplotype show an opposite association. These associations between JC virus antibody response and human leucocyte antigens supports the notion that CD4+ T cells are crucial in the immune defence to JCV and lays the ground for risk stratification for PML and development of therapy and prevention.

## Author Summary

JC virus infection can lead to progressive multifocal leukoencephalopathy in individuals with a compromised immune system, such as during HIV infections or when treated with immunosuppressive or immunomodulating therapies. Progressive multifocal leukoencephalopathy is a rare but potentially fatal disease characterized by progressive damage of the brain white matter at multiple locations. It is therefore of importance to understand the host genetic control of response to JC virus in order to identify patients that can be treated with immunomodulating therapies, common treatments for autoimmune diseases, without increased risk for progressive multifocal leukoencephalopathy. This may also lead to development of preventative or curative anti-JC virus therapies. We here identify genetic variants being associated with JC virus antibody development; a negative association with the human leucocyte antigen DRB1*15-DQA1*01:02-DQB1*06:02 haplotype and a positive association with the DRB1*13-DQA1*01:03-DQB1*06:03 haplotype among controls and patients with multiple sclerosis from Scandinavia. We confirmed the associations in patients with multiple sclerosis from Germany. These associations between JC virus antibody response and human leucocyte antigens imply that CD4+ T cells are crucial in the immune defence and lay the ground for development of therapy and prevention.

## Introduction

Progressive multifocal leukoencephalopathy (PML) was first described neuropathologically during the fifties by Karl Erik Åström [1]. It took until 1971 when JC virus (JCV) was isolated from brain tissue of a patient with PML, since then JCV was accepted as the causative agent of PML [2]. PML used to be a rare demyelinating disease of the central nervous system, mainly seen in patients with lymphoproliferative disease or AIDS. Now several different drugs that interfere with immune functions, such as natalizumab, efalizumab, mycophenolate mofetil, fumaric acid, rituximab, tacrolimus, and possibly azathioprine, cyclosporine and cyclophosphamide have been associated with an increased risk of developing PML. For natalizumab and efalizumab the strongest associations were seen in patients without an underlying disease that predispose for PML itself [3]–[7]. Thus, it is of major importance to develop measures to prevent or treat the condition, including understanding of factors allowing persons to acquire the virus, as carriers, a requisite for later risk for PML.

In patients with multiple sclerosis (MS) treated with natalizumab previous immunosuppressive therapy, an increased duration of therapy, and the positive detection of anti-JCV IgG antibodies as surrogate for the infection with JCV have been established as risk factors for PML [8]–[12]. The anti-JCV antibody status in MS patients is determined by a commercial two step-ELISA. Around 40–50% of the adults are anti-JCV antibody negative [11], [13]–[15]. The cut-off of the commercial assay have been validated in large multicentre cohorts of MS patients with data on JC viruria available, and the false negative rate (sero-negative, but DNA excretion in urine) was estimated with around 2.5% [9]–[11]. In contrast, a recent study that also measured JCV excretion in urine in a comparably small study population (n = 67) indicated a much higher false negative rate of 37%, however, these cases displayed considerably lower JCV DNA copy numbers in the urine. Hypothetically, a vast majority of persons might be exposed to an ubiquitous virus such as JCV, proposed as contamination marker for human excretions, [16] but differ in replicative activity of a persistent asymptomatic infection, and potentially connected to this, the individual level of immune response to the virus. This view would fit with recent serological observations of a continuous anti-JCV reactivity in larger populations, [17] and might imply that actually not the true absence of the JCV infection, but rather the level of the replicative activity of the persistent JCV infection determines the individual risk of developing PML [18]. This risk might then critically depend on host genetic factors that determine the immune response to the virus, and protect from e.g. the spread of the virus from places of peripheral persistency or latency to the brain. Genes of particular interest in this respect are the HLA class I and class II genes where different variants with different peptide presenting abilities may affect the effectiveness of CD4+ and CD8+ T cell immune defence.

Our aim was therefore to test the host genetic regulation of *HLA* genes in the immune response to JCV. We used anti-JCV antibody status and anti-JCV antibody levels as surrogate for the identification of persons carrying a JCV infection in significant and clinically relevant levels and tested association to HLA class I and class II genes.

## Results

Clinical characteristics and demographic data of the included patients and controls are displayed in table 1. Anti-JCV antibody status and levels were determined in the same laboratory for all individuals with an ELISA based method [9].

**Table 1:** Demographic information.

	*Scandinavian MS*	*Swedish controls*	German MS*
Total number genotyped	1621	1064	718
JCV positive (%)	955 (59)	706 (66)	406 (57)
Median JCV nOD in positive individuals	0.398	0.455	0.552
Age at sampling (mean)	39.6±10.6	41.2±11.3	39.3±10.1
% women	72	74	74
Number of individuals with *HLA*-genotypes and serostatus (JCV seropositivity)			
*HLA-A*	1599 (58.9%)	1059 (66.4%)	655 (56.2%)
*HLA-B*	1417 (59.2%)	903 (66.0%)	678 (56.0%)
*HLA-C*	1306 (59.3%)	954 (66.6%)	699 (56.4%)
*HLA-DRB1*	1551 (59.4%)	1059 (66.4%)	631 (55.6%)
*HLA-DQB1*	1452 (58.9%)	963 (66.3%)	698 (56.3%)
*HLA-DQA1*	1409 (58.6%)	908 (65.9%)	690 (56.7%)
With GWAS genotypes (JCV seropositivity)	634 (59.0%)	465 (63.2%)	718 (56.5%)

Demographic information on 1621 Scandinavian MS cases, 1064 Swedish controls and 718 German MS cases with anti-JCV antibody status, anti-JCV nOD antibody levels and HLA-genotypes (from either *HLA-A*, *B*, *C*, *DRB1*, *DQB1*, or *DQA1*). *Since all individuals were GWAS genotyped, they had genotype information for all *HLA*-loci, the numbers shown are the number that passed the quality score ≥0.70 for both alleles for imputed *HLA* genotypes.

**Figure 1:**
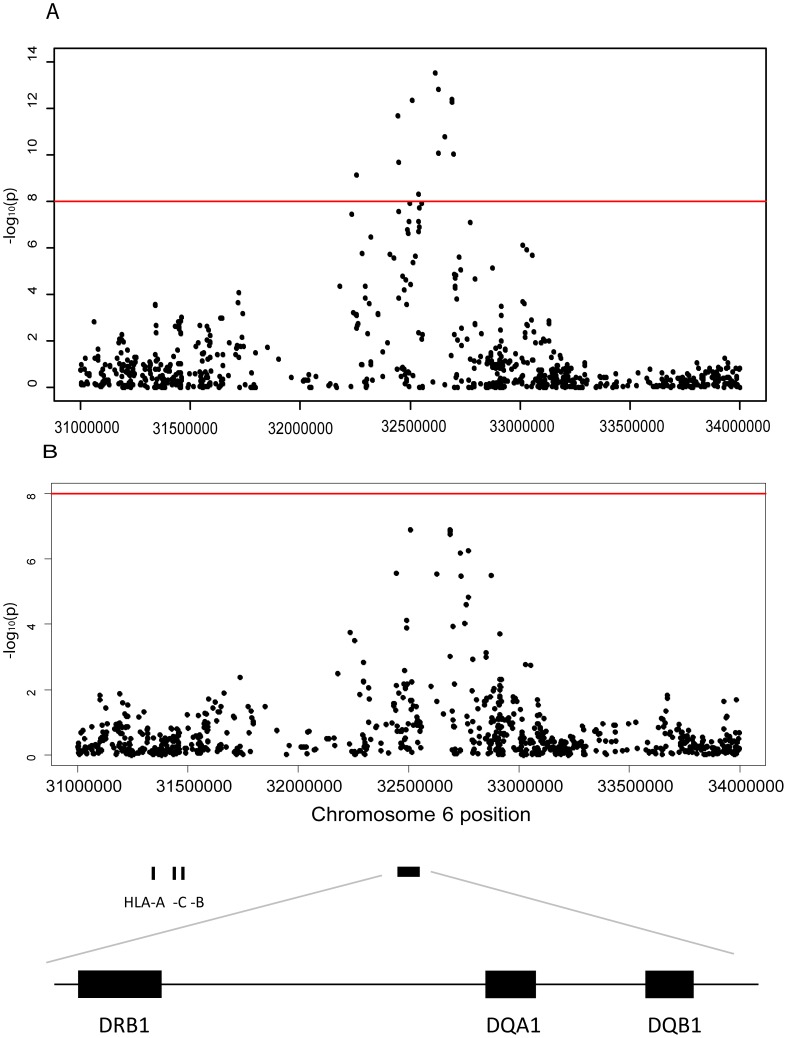
Association between JCA antibody response and markers in the Human Leuococyte region on chromosome 6. **A** Plot of the *HLA* region from the meta-analysis (random effects model) of the association between GWAS markers and JCV serostatus in the Scandinavian (n = 634) and German (n = 718) MS cases and the Swedish controls (n = 465) on chromosome 6. The horizontal line represent a p-values of and 1×10^−8^. All analyses were adjusted for gender, age at sampling, and principal components. The most significant SNP is rs34454237 (p<4×10^−14^) which maps 42.6 kb from the *HLA-DRB1* gene towards the HLA class I genes. **B** Plot of the *HLA* region from the meta-analysis (random effects model) of the association between GWAS markers and transformed anti-JCV nOD levels in the anti-JCV antibody positive Scandinavian (n = 374) and German (n = 294) MS cases and the Swedish controls (n = 406). The horizontal lines represent a p-value of and 1×10^−8^. All analyses adjusted for gender, age at sampling, and principal components. The locations of the *HLA-A*, *-C*, *-B*, *-DRB1*, *-DQA1* and -*DRB1* loci are noted using genome build 36. The most significant SNP is rs3129860 (p<1×10^−7^) which maps 145.7 kb from the *HLA-DRB1* gene in the direction of the *HLA* class I genes.

In this study we selected the HLA complex for scrutiny in view of its potent immune regulatory functions. We performed a meta-analysis of association of markers on chromosome 6 for both anti-JCV antibody status and normalized anti-JCV antibody levels (anti-JCV nOD) of results obtained in the three separate cohorts of individuals shown in table 1. This indicated a strong association signal in the HLA class II region for both anti-JCV antibody status and anti-JCV nOD values (figure 1). The most significant markers for the two analyses (rs34454257 for the anti-JCV antibody status and rs3129860 for anti-JCV nOD values) map 42.6 and 145.7 kb upstream of the *HLA-DRB1* gene in the direction of the HLA class I genes.

With this association signal on chromosome 6p21, it was of interest to determine the particular class II gene variants which were associated. Several HLA-alleles showed association to anti-JCV antibody status in both Scandinavian MS cases and controls (Table 2). The table is organised based on common established extended haplotypes found in the Caucasian population [19]–[21]. It is noteworthy that the DRB1*15-DQA1*01:02-DQB1*06:02-haplotype, the most strongly associated MS genetic risk factor, was found to be negatively associated with the positive detection of anti-JCV antibodies. The OR for DRB1*15 was 0.42 in Scandinavian MS cases and 0.53 in controls. This association was replicated in German MS cases (OR for DRB1*15 0.54 Table 3). Other alleles in this haplotype, DQB1*06:02 and DQA1*01:02, also showed strong protective associations, as expected, since they are in LD with DRB1*15:01. In contrast, the DRB1*13-DQA1*01:03-DQB1*06:03-haplotype was positively associated with anti-JCV antibody status, with an OR = 1.62 in Scandinavian MS cases, OR = 1.55 in Swedish controls (Table 2) and OR = 1.58 in German MS cases (Table 3). In addition the DRB1*03-DQA1*05-DQB1*02 and DQA1*05-DQB1*03:01 haplotypes showed a positive association to anti-JCV antibody status, while the DQA1*05-DQB1*01:01-haplotype was negatively associated with anti-JCV antibody status among controls.

**Table 2:** *HLA*-associations to anti-JCV antibody status in Scandinavian cohort.

	Scandinavian MS cases					Swedish Controls				
	*Frequency (JCV Ab pos/neg)*	*Crude*		Multivariate*		*Frequency (JCV Ab pos/neg)*	*Crude*		Multivariate*	
*Allele*		*p-value*	*OR(95% CI)*	*p-value*	*OR(95% CI)*		*p-value*	*OR(95% CI)*	*p-value*	*OR(95% CI)*
**Haplotype ** DQB1*06:02-DQA1*01:02-DRB1*15-B*07-C*07-A*24										
DQB1*06:02	49.4/70.1	6 e-13	0.44(0.35–0.55)	4 e-10	0.50(0.39–0.64)	22.9/36.3	2 e-6	0.48(0.36–0.65)	6 e-5	0.51(0.37–0.71)
DQA1*01:02	58.4/76.6	6 e-11	0.45(0.35–0.57)	2 e-7	0.51(0.39–0.65)	31.4/47.4	2 e-7	0.46(0.34–0.61)	3 e-5	0.50(0.36–0.69)
DRB1*15	51.9/72.5	7 e-15	0.42(0.33–0.52)	3 e -11	0.45(0.35–0.57)	24.8/36.2	2 e-5	0.53(0.40–0.71)	0.0009	0.61(0.45–0.81)
B*07	39.0/46.7	0.009	0.75(0.60–0.93)	0.03	0.78(0.62–0.97)	27.2/28.3	0.51	0.90		
A*24	16.6/22.2	0.007	0.70(0.55–0.91)	0.02	0.72(0.56–0.93)	14.9/19.9	0.05	0.70(0.55–0.91)	0.05	0.70(0.55–0.91)
**Haplotype ** DQB1*06:03-DQA1*01:03-DRB1*13-B*44-C*?-A*02										
DQB1*06:03	13.9/8.9	0.006	1.63(1.16–2.32)	0.06		20.4/8.6	1 e-5	2.69(1.76–4.24)	3 e-4	2.35(1.51–3.76)
DQA1*01:03	14.2/7.9	5 e-4	1.92(1.34–2.78)	0.008	1.68(1.16–2.47)	21.4/9.7	3 e-5	2.52(1.66–3.93)	0.001	2.16(1.38–3.46)
DRB1*13	23.1/15.5	5 e-4	1.62(1.24–2.13)	0.09		29.2/21.1	0.006	1.55(1.14–2.11)	0.02	1.47(1.07–2.04)
**Haplotype ** DQB1*02-DQA1*05-DRB1*03-B*08-C*07-A*1										
DQB1*02	29.2/23.4	0.02	1.36(1.07–1.74)	0.09		35.7/28.0	0.006	1.52(1.13–2.05)	0.08	
DQA1*05	32.2/24.4	0.003	1.45(1.14–1.85)	0.06		38.5/30.3	0.007	1.52(1.13–2.06)	0.12	
DRB1*03	22.9/18.1	0.03	1.33(1.03–1.73)	0.41		25.3/19.9	0.03	1.42(1.04–1.96)	0.07	
B*08	23.7/18.6	0.02	1.37(1.05–1.79)	0.07		25.2/19.2	0.03	1.47(1.05–2.09)	0.03	1.50(1.07–2.14)
A*01	32.0/26.9	0.03	1.26(1.01–1.57)	0.08		29.4/25.6	0.21	1.21		
**Haplotype ** DQB1*05-DQA1*01:01-DRB1*01-B*07-C*07-A*24										
DQB1*05	20.6/18.5	0.52	1.09			26.6/35.7	0.003	0.64(0.48–0.86)	0.007	0.65(0.47–0.89)
DQA1*01:01	20.2/16.5	0.20	1.20			25.9/33.5	0.02	0.68(0.50–0.92)	0.02	0.66(0.47–0.92)
**Haplotype ** DQB1*03:01-DQA1*05-DRB1*11-B*51-C*05-A*02										
DQB1*03:01	20.2/15.2	0.005	1.48(1.13–1.95)	0.05	1.35(1.01–1.82)	27.3/24.9	0.39	1.15		
DQA1*05	32.2/24.4	0.003	1.45(1.14–1.85)	0.06		38.5/30.3	0.007	1.52(1.13–2.06)	0.12	
C*05	13.8/10.1	0.04	1.45(1.04–2.03)	0.04	1.45(1.04–2.03)	16.9/15.4	0.92	1.02		
B*51	10.8/9.2	0.30	1.21			11.6/6.2	0.02	2.00(1.19–3.49)	0.009	2.04(1.22–3.58)

Results from the association analysis between *HLA*-alleles and JCV seropositivity in the Scandinavian MS cases and the Swedish controls. The frequencies in the second column are the frequencies of *HLA* alleles among JCV Ab seropositive and seronegative respectively. In the crude analysis each allele was analysed on its own, adjusted for gender and age. The analysis was performed in in R version 2.15.1 [50]. The analysis was adjusted for age at sampling and gender. Age at sampling was divided into four categories, 18–29, 30–39, 40–49, and 50 and older, with group 40–49 as the reference. *Multivariate: all nominally significant alleles from the same gene in the same model, adjusted for age and gender.

Common extended *HLA* haplotypes were selected from those published in the literature for the Caucasian population [19]–[21]. Alternative common DRB1*15 haplotypes DQB1*06:02-DQA1*01:02-DRB1*15-B*07-C*07-A*02, DQB1*06:02-DQA1*01:02-DRB1*15-B*07-C*07-A*03, DQB1*06:02-DQA1*01:02-DQB1*15-B*51-C*?-A*02, DQB1*06:02-DQA1*01:02-DRB1*15-B*51-C*?-A*11. The DQB1*03:01-DQA1*05-DRB1*11-B*51-A*02 haplotype exist with many different C alleles, C*05 not being the most common one.

**Table 3:** *HLA*-associations to anti-JCV antibody status among German MS patients.

	*Frequency (JCV Ab pos/neg)*	*Crude*		Multivariate*	
*Allele*		*p-value*	*OR(95% CI)*	*p-value*	*OR(95% CI)*
**Haplotype ** DQB1*06:02-DQA1*01:02-DRB1*15-B*07-C*07-A*24					
DQB1*06:02	49.1/64.7	8 e-5	0.53(0.39–0.73)	4 e-4	0.56(0.40–0.77)
DQA1*01:02	51.3/64.3	0.002	0.60(0.44–0.83)	0.009	0.64(0.46–0.90)
DRB1*15	49.5/64.6	1 e-4	0.54(0.39–0.74)	6 e-4	0.57(0.41–0.78)
B*07	51.1/59.7	0.01	0.65(0.47–0.90)	0.01	0.65(0.47–0.90)
A*24	56.9/56.3	0.63	1.10		
**Haplotype ** DQB1*06:03-DQA1*01:03-DRB1*13-B*44-C*?-A*02					
DRB1*13	63.9/54.7	0.03	1.58(1.06–2.37)	0.14	
Haplotype DQB1*02-DQA1*05-DRB1*03-B*08-C*07-A*1					
DQB1*02	58.6/55.3	0.53	1.11		
DQA1*05	60.8/53.1	0.02	1.45(1.05–2.01)	0.11	
DRB1*03	63.8/54.5	0.07	1.46		
B*08	59.0/55.7	0.21	1.06		
A*01	59.1/55.0	0.52	1.12		
**Haplotype ** DQB1*05-DQA1*01:01-DRB1*01-B*07-C*07-A*24					
DQB1*05	60.8/53.1	0.52	0.89		
DQA1*01:01	54.8/56.9	0.60	0.90		
**Haplotype ** DQB1*03:01-DQA1*05-DRB1*11-B*51-C*05-A*02					
DQB1*03:01	60.6/54.5	0.04	1.43(1.02–2.00)	0.20	
DQA1*05	60.8/53.1	0.02	1.45(1.05–2.01)	0.11	

Results from the association analysis between HLA-alleles and JCV seropositivity in the German MS cases. The frequencies in the second column are the frequencies of HLA alleles among JCV Ab seropositive and seronegative respectively. In the crude analysis each allele was analysed on its own, adjusted for gender and age. The analysis was performed in PLINK 1.07 [49]. The analysis was adjusted for age at sampling, significant principal components from EIGENSTRAT analysis of genomewide SNP data and gender. Age was included as a continuous covariate. *Multivariate: all nominally significant alleles from the same gene in the same model, adjusted for age and gender.

DQB1*06:03 and DQA1*01:03 were not included in the analysis because the allele frequency was below 5%.

Common extended HLA haplotypes were selected from those published in the literature for the Caucasian population [19]–[21]. Alternative common DRB1*15 haplotypes DQB1*06:02-DQA1*01:02-DRB1*15-B*07-C*07-A*02, DQB1*06:02-DQA1*01:02-DRB1*15-B*07-C*07-A*03, DQB1*06:02-DQA1*01:02-DQB1*15-B*51-C*?-A*02, DQB1*06:02-DQA1*01:02-DRB1*15-B*51-C*?-A*11. The DQB1*03:01-DQA1*05-DRB1*11-B*51-A*02 haplotype exist with many different C alleles, C*05 not being the most common one.

The DRB1*15-DQA1*01:02-DQB1*06:02-haplotype also showed an association to lower anti-JCV nOD levels in a linear regression analysis among JCV seropositive individuals, with a significance level of p≤0.001 in the Scandinavian cohorts (Table 4). For DQB1*06:02 beta was between −0.218 and −0.366 in the different cohorts (Table 4 and 5).

**Table 4:** *HLA*-association to transformed JCV nOD levels in Scandinavian cohort.

	Scandinavian MS cases					Swedish Controls				
		Crude		Multivariate*			Crude		Multivariate*	
*Allele*	*Median nOD JCV Ab (pos/neg)*†	*p-value*	*beta*	*p-value*	*beta*	*Median nOD JCV Ab (pos/neg)*†	*p-value*	*beta*	*p-value*	*beta*
Haplotype DQB1*06:02-DQA1*01:02-DRB1*15-B*07-C*07-A*24										
DQB1*06:02	0.357/0.453	**0.001**	−0.218	**0.001**	−0.218	0.322/0.488	**8 e-5**	−0.366	**0.0004**	−0.333
DQA1*01:02	0.360/0.476	**0.001**	−0.233	**0.001**	−0.233	0.372/0.492	**0.004**	−0.252	**0.02**	−0.149
DRB1*15	0.356/0.452	**0.001**	−0.238	**0.006**	−0.188	0.308/0.482	**5 e-6**	−0.399	**5 e-6**	−0.399
**Haplotype ** DQB1*06:03-DQA1*01:03-DRB1*13-B*44-C*?-A*02										
DRB1*13	0.495/0.363	**0.02**	0.197	0.08	0.139	0.459/0.450	0.24	0.098		
**Haplotype ** DQB1*03:01-DQA1*05-DRB1*11-B*51-C*05-A*02										
DQB1*03:01	0.476/0.398	0.08	0.153			0.523/0.427	**0.05**	0.171	0.06	0.151
DQA1*05	0.412/0.399	0.11	0.150			0.528/0.421	**0.04**	0.172	0.16	0.087

Results from the linear regression analysis of the association between HLA-alleles and JCV nOD levels. Only alleles that are nominally significant (p<0.05) alleles in any cohort in this or table 5 are presented. P-values that reach nominal significance, 0.05 are marked in bold. The median nOD levels are given among individuals positive or negative for respective *HLA* allele. In the crude analysis each allele was analysed on its own, adjusted for gender and age. Age at sampling was divided into four categories, 18–29, 30–39, 40–49, and 50 and older, with group 40–49 as the reference. The analysis was performed in R version 2.15.1 [50]. *Multivariate: all nominally significant alleles from the same gene in the same model, adjusted for age and gender. † Median nOD is given among individuals positive or negative for respective *HLA* allele.

Common extended HLA haplotypes were selected from those published in the literature for the Caucasian population [19]–[21]. Alternative common DRB1*15 haplotypes DQB1*06:02-DQA1*01:02-DRB1*15-B*07-C*07-A*02, DQB1*06:02-DQA1*01:02-DRB1*15-B*07-C*07-A*03, DQB1*06:02-DQA1*01:02-DRB1*15-B*51-C*?-A*02, DQB1*06:02-DQA1*01:02-DRB1*15-B*51-C*?-A*11. The DQB1*03:01-DQA1*05-DRB1*11-B*51-A*02 haplotype exist with many different C alleles, C*05 not being the most common one.

**Table 5:** *HLA*-association to transformed JCV nOD levels in German MS patients.

Allele	Median *nOD JCV Ab (pos/neg)*	*p-value*	*beta*
**Haplotype ** DQB1*06:02-DQA1*01:02-DRB1*15-B*07-C*07-A*24			
DQB1*06:02	0.480/0.598	**0.007**	−0.276
DQA1*01:02	0.508/0.633	**0.01**	−0.256
DRB1*15	0.506/0.596	**0.04**	−0.213
**Haplotype ** DQB1*06:03-DQA1*01:03-DRB1*13-B*44-C*?-A*02			
DRB1*13	0.471/0.572	0.18	−0.168
**Haplotype ** DQB1*03:01-DQA1*05-DRB1*11-B*51-C*05-A*02			
DQB1*03:01	0.634/0.535	0.07	0.200
DQA1*05	0.596/0.526	0.48	0.073

Results from the linear regression analysis of the association between *HLA*-alleles and JCV nOD levels. Only alleles that are nominally significant (p<0.05) alleles in any cohort in this or table 4 are presented. P-values that reach nominal significance, 0.05 are marked in bold. The median nOD levels are given among individuals positive or negative for respective HLA allele. Each allele was analysed on its own, adjusted for age at sampling, significant principal components from EIGENSTRAT analysis of genomewide SNP data and gender. Age was included as a continuous covariate. The analysis was carried out in PLINK 1.07 [49]. As only alleles on the DQB1*06:02-DQA1*01:02-DRB1*15 haplotype were significant no multivariate analysis was performed. * Median nOD is given among individuals positive or negative for respective *HLA* allele.

Common extended HLA haplotypes were selected from those published in the literature for the Caucasian population [19]–[21]. Alternative common DRB1*15 haplotypes DQB1*06:02-DQA1*01:02-DRB1*15-B*07-C*07-A*02, DQB1*06:02-DQA1*01:02-DRB1*15-B*07-C*07-A*03, DQB1*06:02-DQA1*01:02-DRB1*15-B*51-C*?-A*02, DQB1*06:02-DQA1*01:02-DRB1*15-B*51-C*?-A*11. The DQB1*03:01-DQA1*05-DRB1*11-B*51-A*02 haplotype exist with many different C alleles, C*05 not being the most common one.

DRB1*13 showed an association to higher anti JCV nOD levels among Scandinavian MS cases, p = 0.02, beta = 0.197, but not in German MS cases or Swedish controls (Table 4 and 5). In addition DQB1*03:01 and DQA1*05 showed an association to higher transformed anti-JCV nOD levels among Swedish controls (Table 4).

Most of the HLA associations to both anti-JCV antibody status and anti-JCV nOD levels remained similar when other nominally associated HLA alleles for the same gene are included in the regression analysis (Table 2 to 5).

**Figure 2:**
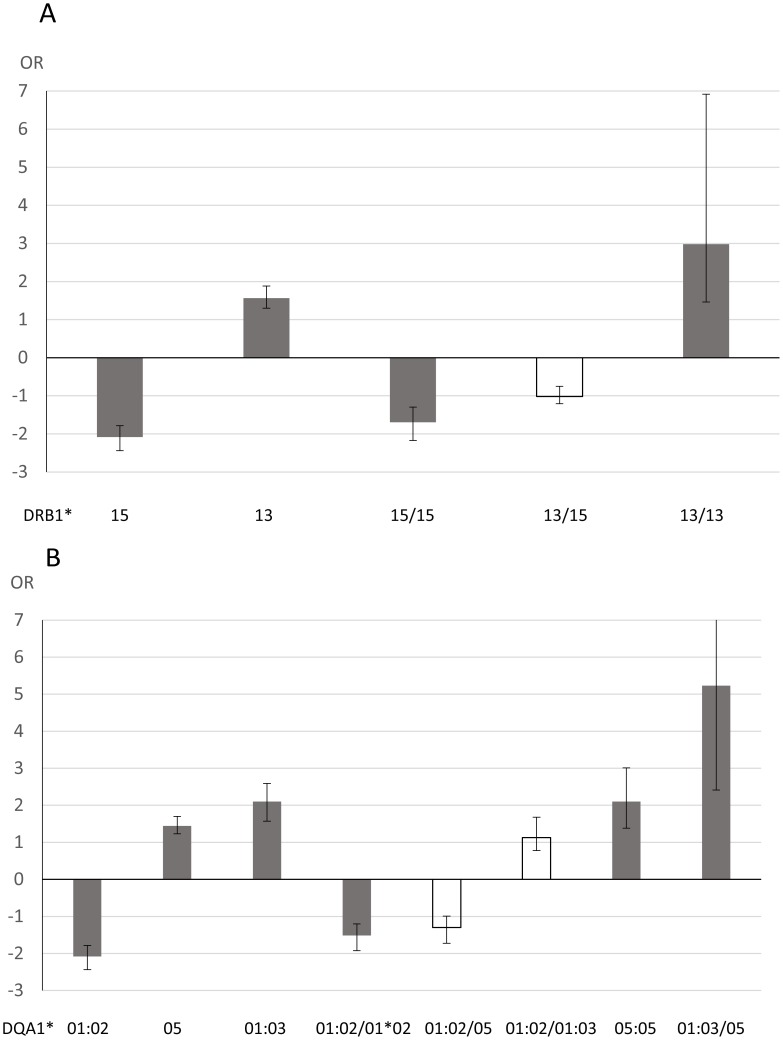
Analysis of association between *HLA* genotypes and anti-JCV antibody status in joint analysis of Swedish controls, Scandinavian and German MS patients. **A** Odds ratio (OR) for *DRB1* alleles and genotypes from logistic regression analyses performed in R version 2.15.1 [50]. The analyses were adjusted for gender, cohort (Swedish controls, Scandinavian MS patients or German MS patients) and age at sampling. Age at sampling was divided into four categories, 18–29, 30–39, 40–49, and 50 and older, with group 40–49 as the reference. Error bars represents 95% confidence intervals. OR below 1 are plotted as −1/OR. Grey indicates associations with p<0.05, white p>0.05. **B** Odds ratio (OR) for *DRB1* alleles and genotypes from logistic regression analyses performed in R version 2.15.1 [50]. The analyses were adjusted for gender, cohort (Swedish controls, Scandinavian MS patients or German MS patients) and age at sampling. Age at sampling was divided into four categories, 18–29, 30–39, 40–49, and 50 and older, with group 40–49 as the reference. Error bars represents 95% confidence intervals. OR below 1 are plotted as −1/OR. Grey indicates associations with p<0.05, white p>0.05. DQA1*01:03 homozygotes are not included as this combination was so rare (0.4%).

The OR for the association of the presence of DRB1*15 and for DRB1*15 homozygotes for JCV antibody status did not differ, suggestive of a dominant DRB1*15 effect (Figure 2A). Conversely, the DRB1*13 homozygotes showed a slightly stronger association compared to presence of DRB1*13, although the 95%CI do overlap. DRB1*13/15 heterozygotes were not significantly associated with JCV seropositivity indicating that the effect of the two haplotypes counteract each other. Similar results were seen for the DQA1 locus (Figure 2B), but here the DQA1*01:03/05 heterozygotes are associated with an OR as high as 5.23.

Consistent with the effect on the qualitative anti-JCV status, the DRB1*15 haplotype appeared to act dominantly also on anti-JCV nOD levels as presence of DRB1*15 showed a similar association to DRB1*15 homozygotes, while the DRB1*11 haplotype had an additive effect (Table 6). The effect of these two haplotypes cancel each other out as DRB1*11/15 heterozygotes showed no association to anti-JCV nOD levels.

**Table 6:** Association of *HLA* genotypes to transformed JCV nOD levels in joint analysis of Swedish controls and Scandinavian and German MS patients.

***Allele/genotype***	*Median nOD (pos/neg)* *	*p-value*	*beta*
*DRB1* * *15*	0.364/0.487	4 e-9	−0.189
*DRB1* * *11*	0.601/0.428	0.008	0.142
*DRB1* * *11/11*	1.037/0.442	0.04	0.769
*DRB1* * *11/15*	0.504/0.442	0.94	0.007
*DRB1* * *15/15*	0.329/0.456	0.005	−0.181

Results from the linear regression analysis of the association between HLA-alleles and genotypes and JCV nOD levels. The median nOD levels are given among individuals positive or negative for respective HLA allele or genotype. The analyses were performed in R version 2.15.1 [50] and were adjusted for gender, cohort (Swedish controls, Scandinavian MS patients or German MS patients) and age (as a continuous covariate).

* Median nOD is given among individuals positive or negative for respective HLA allele or genotype.

We reanalysed the association of SNPs on chromosome 6 to anti-JCV antibody status and anti-JCV nOD levels when including all *HLA* alleles that remained associated in the multivariate analysis as covariates. This lead to an almost complete abolishment of the association peak on chromosome 6, with the most significant remaining associations being p = 0.0001 for a handful of markers (data not shown). This indicates that the association we observed in the *HLA* region was almost completely explained by the *HLA* alleles listed in tables 2–4.

## Discussion

We here demonstrate a host genetic HLA complex mediated influence on anti-JCV antibody status and anti-JCV antibody levels as surrogate for the susceptibility of the infection with JCV or the activity of the infection with JCV, respectively.

We report a strong negative association with anti-JCV antibody positivity, and to a lesser extent, to anti-JCV nOD levels, for the HLA-DRB1*15-DQA1*01:02-DQB1*06:02-haplotype in all three datasets. In contrast, the DRB1*13-DQA1*01:03-DQB1*06:03-haplotype is associated to increased signs of JCV carriage as assessed serologically. We further find that the DRB1*15-DQA1*01:02-DQB1*06:02 haplotype acts dominantly, one copy being sufficient to reduce the ability to form anti-JCV antibodies while the DRB1*13-DQA1*01:03-DQB1*06:03 haplotype acts in an additive fashion. Neither of the haplotypes dominates over the other. A recent study demonstrated considerable variation in which JCV peptides were recognized by T cells [22]. A most straight forward interpretation of the present findings is that the DRB1*15:01 haplotype displays class II molecules that are especially able to present JCV antigens/peptides that are instrumental in activating CD4+ T cells that support the elimination or control of the virus upon exposure to the host. Hence, the opposite would be valid for the haplotype associated with increased carriage of the JCV. Thus hypothetically a large proportion of those persons being sero-negative might have encountered the virus, but had an efficient immune response following primary infection, with low viral turnover or the lack of viral persistency, and low anti-JCV IgG as consequence.

Recent serological studies support such a concept: antibody reactivity as measured by ELISA resembles a continuum from non-reactive to highly reactive in particular in persons not excreting the virus in urine [9]. This led to the introduction of a second-step confirmation test when determining the anti-JCV sero-status. However, this pattern of continuous reactivity is also seen with alternative assay formats, which suggest that a vast majority of persons have been exposed to JCV, but have a level of the antibody response to JCV below the assay cut-off, possibly due to an efficient control of the virus with low viral turn-over [13],[17]. This would also explain the higher false negative rate of serological studies observed in recent publications [23], [24].

Although CD8+ cells, restricted by class I molecules are critical in eliminating virus infected cells, antigen specific CD4+ *HLA* class II restricted cells are crucial for providing T cell help through a variety of cytokines and activation of antigen presenting dendritic cells [25]. The findings may pave the way for finding epitopes in JCV critical for immune defence which could impact on vaccination strategies. Any direct clinical implications of the data, or use, for example in risk stratifications, remain to be determined.

The DRB1*13-DQA1*01:03-DQB1*06:03-haplotype shows a positive association to anti-JCV antibody serostatus, and was also to higher anti-JCV nOD levels. Hypothetically, a less effective viral immune control with higher viral turnover may be consistent with a chronically higher stimulation of the B cell arm of the immunity resulting in higher antibody levels. This might help us understand why patients that develop PLM during therapy with natalizumab had increased anti-JCV antibody levels already prior to development of PML, and why it might be rational to include the level of the anti-JCV response into PML risk stratification strategies [26].

Recent data suggests that PML-specific viral mutations are acquired intra-individually e.g. in the *VP1*-region and the regulatory region of the viral genome [27], [28]. It is tempting to speculate that viral PML-specific mutations, although being a random event, are more likely to occur in persons with an inefficient control of the infection with JCV. The host genetic data presented here might therefore be a first step helping to understand how the interplay of host- and viral genetic factors might lead to the development of PML in some, but not all persons exposed to certain immunosuppressive therapies. Our study is however lacking a sufficient number of cases of PML and is therefore not designed and empowered to test this directly.

There is one previous paper studying the *HLA* association to PLM [29]. In this paper 123 Caucasian PML cases, the majority whom were HIV positive, were compared with a large group of HIV positive individuals. The study was limited to the association of HLA class I antigens. While *A3* was found to be nominally negatively associated with PML, *B18* was found to be positively associated. We do not find any of these alleles associated with anti-JCV antibody formation in our study. However, the *A3* association to PML possibly is explained by the same effect as the DRB1*15-DQA1*01:02-DQB1*06:02 association we see in our study, considering that *A3* can be present on the same extended haplotype. Studies in larger cohorts of PML patients with appropriate controls testing the association of class II antigens are warranted. A recent investigation has studied the stimulation of CD4+ T cells by pools of JCV peptides among healthy donors with different *HLA-DRB1* alleles [22]. For haplotypes where we see an increased OR for sero-status and positive correlation to JCV-Ab levels (DRB1*13 and DRB1*03) they observe reduced stimulation of CD4+ cells, while the opposite was true for DRB1*15. Hence, both antibody response and T-cell response to JCV infection are affected by *HLA*-class II antigens, which is consistent with our observations of potent *HLA* class II gene variant effects in large cohorts of persons.

HLA associations to some viral infections have been seen previously. The HLA class II genes were recently reported as host genetic factors influencing the IgG response to EBNA1, an Epstein Barr virus-related protein [30]. In addition, there are well documented class II allelic influences on Hepatitis C [31] and recently a highly associated SNP in the *HLA* region was demonstrated in relation to human papilloma virus infection [32]. *HLA* class II associations have also been seen in chronic hepatitis B infections as well as response to hepatitis B vaccination [33]–[35]. Association to the HLA class I related *MICB* gene have also been reported to hypovolemic shock caused by dengue viral infection and HIV viral load [36], [37]. Another MIC gene, *MICA* has been associated to hepatitis C virus induced hepato cellular carcinoma [38]. In our data, after adjusting the association for HLA class II associated alleles the most strongly associated marker in the class I region is rs3094014 (p<0.02) which is in LD with both the *MICB* (r^2^ 0.76 for rs3132468 associated with dengue fever) and *MICA* (r^2^ 0.90 for rs2596542 reported to be associated with hepatitis C virus induced hepato cellular carcinoma) using European 1000 genomes and HapMap data and may therefore represent the same signal.

Interestingly, the DRB1*15 haplotype is also the most strongly associated genetic risk factor for MS [39]. Consistent with this, the demographic data in our study suggests that anti-JCV antibody positivity is somewhat lower among MS cases (59%), compared to controls (66%, p = 0.02). A protection against the establishment of a persistent JCV infection with positive detection of anti-JCV antibodies provided by the DRB1*15 haplotype would then explain the lower sero-prevalence among cases. The presence of an association with the DRB1*15-haplotype in controls also indicates that the association is not likely due to an aberrant immune response to JCV infection in MS cases.

In conclusion, we here demonstrate strong associations of class II gene variants on JCV infection. Hence, CD4+ T cells, restricted by class II molecules are crucial in the host control of JCV infection. Our data is of importance for a better understanding of JCV infection and virus-host interactions, and might pave the way for new developments for an improved PML risk stratification, and preventive or curative future anti-JCV therapies.

## Materials and Methods

### Ethics statement

The study was approved by the regional ethical committees in each country involved; Stockholm regional Ethical Review Board (Sweden), the Ethical review boards at the Heinrich-Heine Universität Düsseldorf and the Technische Universität München (Germany) and the Danish Ethical Committee Review Board for Copenhagen and Frederiksberg (Denmark). All participants provided written informed consent.

### Patients and controls

A Scandinavian dataset consisting of 2015 Swedish persons with MS from two separate studies EIMS [40] and IMSE [41] with 1259 population based controls, and 157 Danish MS patients treated with natalizumab in Copenhagen. *HLA*-genotypes and anti-JCV antibody status were available for 1621 MS cases and 1064 controls (table 1).

A German dataset of 745 MS patients, 718 with GWAS data, was used for replication. The cohort was recruited from multiple sites in Germany and included persons treated with interferon-beta for at least 6 months. GWAS genotyping for these MS cases had been performed in the same laboratory with the same chip as the Scandinavian datasets.

### *HLA* and SNP-typing

*HLA*-genotypes came from three different sources. Low resolution Sequence Specific amplification (Olerup, Saltsjöbaden, Sweden) [42] were for genotyping of 2115 Swedish cases and controls for *HLA-A*, 2140 for *HLA-DRB1*, and 161 for *HLA-C*. For *HLA-B* a Luminex based reverse PCR-SSO (One Lambda, Inc., Canoga Park, CA, USA) was used for 173 persons [39]. And finally imputation either using HLA*IMP [43] with genotypes from the IMSGC WTCCC2 MS GWAS, [44] or with HLA*IMP:2 [45] using genotypes from the Immunochip [46] was used. The former was used for both the Danish and German cohort while both were used in the Swedish cohort. Imputed HLA data was available for 1105 Swedish persons from the IMSGC WTCCC2 and for 2220 Swedish persons using Immunochip genotypes. In cases where the genotypes for any individual were discordant between platforms, the following order of precedence was used: classical, Immunochip imputed, GWAS imputed. A quality value for allele probability of 0.7 was used as a threshold for imputed HLA genotypes.

In this study we used SNP genotypes from MS GWAS study to analyse association to the HLA region [44]. Genome-wide SNP markers were genotyped as part of the IMSGC WTCCC2 MS GWAS on the Human660-Quad chip, and genotype calling and markers were quality controlled as previously described [44].

Cases with previous intravenous IgG treatment were excluded. In the Scandinavian cohort all persons were of Scandinavian ancestry, and all MS cases fulfilled the McDonald or Poser criteria for MS. For the German MS cases, a total number of 749 cases were GWAs genotyped; 25 persons were removed as they were outliers in the principal component analysis (PCA), 4 were removed due to unsuccessful genotyping, and 2 because of natalizumab treatment at blood draw.

### Anti-JCV antibody determination

JCV serology response was determined from plasma or serum using a two-step assay, [9] performed at Focus Diagnostics (Cypress, CA, USA) and sponsored by Biogen Idec (Cambridge, MA, USA). In the first step of the ELISA assay optical density (OD) were measured. Samples with OD>0.25 were considered positive while samples with OD<0.10 were considered negative. For samples in the intermediary interval (0.10–0.25) a second assay step was used to determine the percentage of inhibition during a pre-incubation with soluble JCV-like particles. Samples in this intermediary interval with an inhibition >40% were considered positive while those with an inhibition <40% were classified as negative.

The assay has been estimated to have a false negative rate for JCV carriage of 2–3%. For the quantitative analysis, normalised OD values (nOD) from the first step ELISA were transformed using rank based transformation in the GenABEL-package in R [47].

### Statistical analysis

#### Association of GWAS-markers on chromosome 6 to anti-viral antibodies

To generate principal components and control for population stratification, an EIGENSTRAT analysis was performed in each cohort separately, using Eigensoft [48]. This analysis was performed after SNP-pruning with r^2^>0.2 were removed using PLINK 1.07 [49]. All principal components with a p-value below 0.05 were included as covariates in the regression models. All persons considered as outliers in the PCA were removed.

Logistic regression analysis (for anti-JCV antibody status) and linear regression analysis (for transformed anti–JCV nOD-levels among JCV seropositive individuals) were performed in PLINK 1.07, adjusting for age, gender, and principal components. The threshold for Hardy-Weinberg equilibrium test was p>0.001, and minor allele frequency >0.05. We also ran the same analysis, where we adjusted for all *HLA*-alleles associated with each outcome, with p<0.05 in the final model as cut-off for inclusion.

A meta-analysis of the results from the different cohorts was performed in PLINK 1.07, using both a fixed and a random effects model.

#### Analysis of *HLA*-association

MS cases and controls were analysed separately. Association to anti-JCV antibodies was tested separately for alleles with a frequency higher than 5% separately. As a second step, all nominally significant alleles from the same *HLA*-gene were analysed in a multivariate regression model. Association to anti-JCV serostatus was performed with logistic regression, and association to transformed anti-JCV nOD-values was performed with linear regression, in R [50] or PLINK 1.07 [49]. We adjusted for age at sampling and gender in all analyses.

For the German dataset, it was also possible to adjust for principal components, but for the Scandinavian dataset, this was not possible since the majority of samples were not genotyped with genome wide markers.

For the analysis of effect of genotypes a joint analysis was performed including all cohorts, in this analysis covariates for age at sampling, gender and cohort were included.

Analysis of the data was carried out by Emilie Sundqvist, Eva Albrecht and Ingrid Kockum.
