# Correction: JC Polyomavirus Infection Is Strongly Controlled by Human Leucocyte Antigen Class II Variants

**DOI:** 10.1371/journal.ppat.1004430

**Published:** 2014-09-08

**Authors:** The PLOS Pathogens Staff

The name of the third to last author in the authorship listing is spelled incorrectly. The correct spelling of the name is: Bernhard Hemmer. The citation from the original article remains the same.
